# Combining genetic and distributional approaches to sourcing introduced species: a case study on the Nile monitor (*Varanus niloticus*) in Florida

**DOI:** 10.1098/rsos.150619

**Published:** 2016-04-20

**Authors:** Stephanie A. Dowell, Jared P. Wood, Todd S. Campbell, Sergios-Orestis Kolokotronis, Evon R. Hekkala

**Affiliations:** 1 Department of Biological Sciences, Fordham University, 441 East Fordham Road, Bronx, NY 10458, USA; 2 Department of Biology, University of Louisville, 2301 South 3rd Street, Louisville, KY 40292, USA; 3 Department of Biology, University of Tampa, 401 West Kennedy Boulevard, Tampa, FL 33606, USA

**Keywords:** invasive species, source population, introduction pathway, pet trade, DNA assignment, ecological niche model

## Abstract

Three separate breeding populations of the Nile monitor (*Varanus niloticus*) have been identified in Florida, USA, located in Cape Coral, West Palm Beach and Homestead Air Reserve Base. This large, predatory lizard could have negative effects on Florida's native wildlife. Here, we infer the source of the introduced populations using genetic and statistical approaches, as well as estimate the potential non-native distribution of *V. niloticus* in North America. We collected genetic data from 25 Florida individuals as well as utilized genetic datasets from reference individuals spanning the full native distribution throughout sub-Saharan Africa. Using occurrence data from the inferred source population and the full species range, we built ecological niche models (ENMs) and projected them onto North America to determine regions with suitable climate. Our results indicated that the introduced populations resulted from three separate introduction events, and all originated from the southern coastal region of West Africa. The ENM built from the West African source population predicted only the southernmost portions of North America to be suitable. Conversely, the model derived from the full species’ range predicted suitable climates across a large portion of the United States. This information can be used to focus management and eradication efforts.

## 1. Introduction

The prevalence of introduced species continues to rise as a consequence of globalization and expanding human transportation [1,2]. While a majority of non-native organisms fail to survive and become established [3], those that do can cause severe harm to native ecosystems [4,5]. Invasive alien species are considered the second leading cause of biodiversity loss, preceded only by anthropogenic habitat destruction [6,7]. Negative effects of introduced species on native populations often include competition for resources [8], predation of native species [9], habitat alteration [10], and the introduction of novel parasites and diseases [11]. In many cases, the presence of an introduced species has resulted in extreme consequences, such as complete restructuring of the food web and drastic changes in community composition [4,5]. Florida, USA, has been disproportionately affected by introduced organisms, supporting the highest number of established non-native herpetofauna in the world [12]. The cumulative effects of introduced alien species have placed an enormous burden on the fragile Florida ecosystem [13].

**Figure 1.**
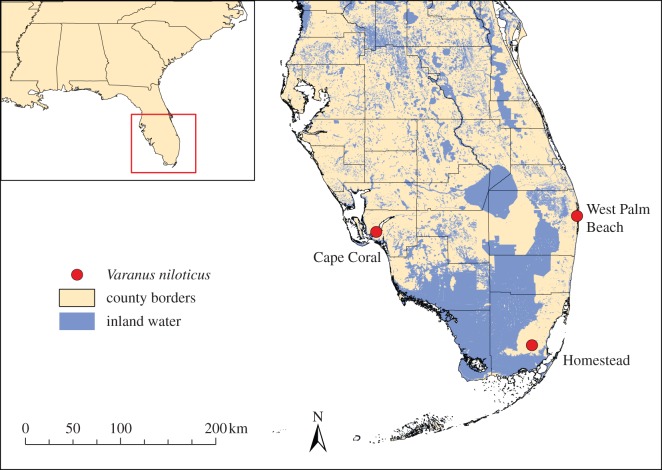
Map of Florida State, USA, showing the location of the introduced *Varanus niloticus* populations.

The Nile monitor lizard, *Varanus niloticus*, was added to Florida's long list of introduced species in 1990, when individuals were first observed in the Cape Coral region [14]. Currently, three separate breeding populations have been documented, inhabiting Cape Coral, West Palm Beach and the Homestead Air Reserve Base (figure 1) [15,16]. The largest of these populations, located in Cape Coral, is estimated to comprise over 1000 individuals [15], whereas the other populations are smaller. To date, there have been 80 and 47 confirmed *V. niloticus* sightings in West Palm Beach and Homestead, respectively, compared with 389 public sightings [16] and over 400 captures in Cape Coral (by T.S.C. and the City of Cape Coral). This lizard species is native to sub-Saharan Africa and occurs throughout most of the continent, occupying a broad range of habitats and vegetation zones [17,18]. Owing to its semi-aquatic nature, *V. niloticus* is largely dependent on permanent bodies of water throughout its native distribution [17,18], and in Florida they are commonly observed along canal banks, tidal creeks and mangroves [15]. This species is a generalist predator with diet preferences ranging from molluscs and amphibians to birds and small mammals [17]. Because of their large size, reaching 2 m in length, *V. niloticus* represents a severe threat to Florida wildlife, able to prey upon a variety of species and quickly outgrowing potential predators [14]. Other life-history characteristics of *V. niloticus*, including early sexual maturation (around 2 years of age), high fecundity (laying up to 60 eggs in a single clutch) and high motility (occupying home ranges up to 50 km^2^), are indicative of a high invasive potential [17,19].

The introduction history of the *V. niloticus* populations has not been confirmed; however, the pet trade likely played a dominant role. Approximately 84% of the introduced amphibian and reptile species in Florida have resulted from the pet industry [12]. Nile monitors are frequently imported for pets and constitute 22.9% of the global trade of live varanids [20]. A few theories exists surrounding the details of the *V. niloticus* introduction in Florida. One theory suggests that individual pets escaped or were released by owners when the animals became too difficult to manage [14,15]. The other theory posits that *V. niloticus* individuals were released by reptile dealers in order to establish a breeding population in which to capture individuals for resale, thus avoiding the costs of husbandry or international trade [14,15].

A recent genetic analysis of native *V. niloticus* populations throughout Africa identified three geographically structured lineages [21]. This study proposed that the westernmost population be considered a separate species, based on the large degree of genetic divergence and low level of admixture [21]. The other genetic lineages, partitioned into a northern and a southern group, also displayed moderate levels of genetic differentiation [21]. Restricted gene flow among populations under differing environmental conditions can result in local adaptation, where local genotypes have a fitness advantage over genotypes originating from other environments [22–25]. Evidence of local adaptation has been documented in a variety of herpetofauna, including lizards [26–28] and amphibians [24,29,30]. Because of the considerable environmental variation across the African continent, and the likelihood of multiple cryptic species within *V. niloticus*, understanding the origin of the introduced populations is of utmost importance in predicting their spread.

Genetic methods can be used to understand the source of introduced organisms and to estimate the number of introduction events. These approaches rely on first characterizing the genetic variation across the species’ native range. One method uses DNA sequence data to establish the genealogical relationships among the introduced individuals and reference individuals from known localities throughout the native range [31]. In this approach, the introduction pathway can be reconstructed based on geographical information and ancestry [31–33]. The second method typically relies on microsatellite data and assigns individuals to a likely source population by comparing allele frequencies across an array of native populations [31,34,35]. Both types of analyses are dependent on the degree of genetic partitioning present among native populations. For organisms with a large degree of genetic structure, it is possible to assign introduced or unknown individuals to a more precise geographical origin than for organisms that are genetically homogeneous [31].

Evidence suggests that the *V. niloticus* population in Cape Coral is expanding into new regions [15], and therefore, increased efforts are required to effectively control the populations and prevent additional introductions from occurring. In this study, we identify the geographical origin of the introduced *V. niloticus* populations using both genealogical methods and frequency-based approaches. Furthermore, we estimate the number of independent introduction events that have led to the current *V. niloticus* populations in Florida. Because the hypothesis of local adaptation among the *V. niloticus* lineages has not been tested, we predict suitable climates in North America under both assumptions: (i) local adaptation among populations and (ii) constant fitness levels across the full range of the species.

## 2. Material and methods

### 2.1. Sample collection and laboratory protocols

We obtained a total of 25 tissue samples from the introduced *V. niloticus* populations, consisting of 15 individuals from Cape Coral, 5 from West Palm Beach and 5 from Homestead. Individuals were collected from Cape Coral by trapping [15], and tissue samples from West Palm Beach and Homestead were provided by the Florida Wildlife Commission, Environmental Flight of Homestead Air Reserve Base and USDA-APHIS. All samples were collected between 2006 and 2013. DNA extraction from muscle tissue was carried out using the DNeasy Blood and Tissue Kit (Qiagen), following the manufacturer's protocol for tissue. We examined four mitochondrial loci, including 12S rRNA (293 bp), *ND1* (457 bp), *ND2* (528 bp) and *ND4* (816 bp); as well as three nuclear loci, *RAG-1* (781 bp), *KIAA1217* (595 bp) and *KIAA1549* (781 bp), totaling 4251 bp. PCR amplification was carried out in 21 µl reactions and conditions followed those of Dowell *et al.* [21]. Amplification success was confirmed on a 1.5% agarose gel, and PCR products were sequenced using the Sanger method at Macrogen (New York, NY, USA). Forward and reverse chromatograms were edited and assembled into contigs in Sequencher v 4.1 (Gene Codes Corp.). All generated sequences were deposited in GenBank under the accession numbers KT894381–KT894551.

We amplified 11 microsatellite loci originally developed for *Varanus komodoensis*—*K7*, *K10*, *K11*, *K15*, *K22* and *K23* [36,37]; *V. salvator*—*VARSA10* and *VARSA07* [38]; and *V. acanthurus*—*VA17*, *VA38* and *VA74* [39], following the modifications of Dowell *et al.* [40]. We carried out fragment analysis on an ABI 3100 Genetic Analyzer (Applied Biosystems, Inc.) with GeneScan 500 LIZ size standard, and genotypes were scored with GeneMarker (SoftGenetics).

We additionally used the recent dataset of Dowell *et al.* [21], totalling 125 samples of *V. niloticus* originating from throughout their native distribution (see electronic supplementary material, S1 for detailed locality information). These reference samples from known localities across Africa included both contemporary samples and historic museum specimens [21]. The DNA sequence dataset consisted of the mitochondrial and nuclear loci detailed above (GenBank accession numbers JN673347, JN673350–JN673352, JN673363–JN673365 and KT720497–KT721289). The reference microsatellite dataset was composed of all 11 loci for contemporary samples (*N* = 67) and five loci for museum samples (*N* = 42), including *K7*, *K11*, *VARSA10*, *VA38* and *VA74* (see electronic supplementary material, S2 for genotypes). This discrepancy in the number of loci was due to a lower amplification success for museum specimens [21].

### 2.2. Phylogenetic and genealogical approaches

DNA sequences from the introduced populations were analysed together with sequences from reference individuals across the species’ native range in Africa. We aligned all DNA sequences using Clustal-W [41] implemented in MEGA 6 [42]. Haplotype reconstruction of nuclear gene regions was carried out using the PHASE [43,44] and FastPHASE [45] algorithms implemented in DnaSP 5.10.1 [46].

To infer the source lineage(s) of the introduced *V. niloticus* individuals, DNA sequence data from reference and Florida individuals were analysed using maximum-likelihood (ML) with RAxML 8.1.15 [47]. We analysed the full concatenated dataset, partitioned by gene region, under the general-time-reversible substitution model [48] with rate heterogeneity across sites modelled by a *Γ* distribution and four discrete rate categories [49]. We implemented 100 ML tree searches starting each time with a random stepwise addition maximum-parsimony tree, followed by 1000 bootstrap replicates to assess node robustness [50]. To confirm that the number of bootstraps was sufficient to achieve stable support values, we conducted *a posteriori* bootstrap convergence tests with the frequency-based and majority-rule consensus criteria, as implemented in RAxML [51].

To examine the level of connectivity among introduced and reference haplotypes, indicative of the source population as well as the number of introduction events, we constructed a minimum-spanning haplotype network in TCS 1.21 [52] using the mitochondrial dataset (2094 bp) and the default threshold of 95% parsimony. If all Florida haplotypes are connected to reference haplotypes by a single branch, implying monophyly, this would suggest that all haplotypes could have resulted from a single introduction event. However, if Florida haplotypes are interspersed throughout the network of reference haplotypes, then multiple introduction events would be likely.

### 2.3. Frequency-based approaches

We further examined the origin of the introduced *V. niloticus* populations by conducting assignment tests with the microsatellite data. We assigned introduced and reference individuals to populations with GeneClass 2.0 [53] using the Bayesian approach described by Rannala & Mountain [54] which has been shown to outperform other assignment methods [55]. Probabilities were computed with the Paetkau *et al.* [56] algorithm based on 100 000 simulations and an *α* level of 0.01.

We also estimated the geographical origin of the Florida *V. niloticus* individuals with the continuous assignment method (CAM) implemented in SCAT 2.0.1 [57]. This program uses a Bayesian approach to assign individuals to a geographical origin and the CAM method can assign samples to localities anywhere in the study region, independently of reference sampling sites [57]. This is accomplished by generating a spatial gradient of microsatellite allele frequencies from georeferenced genotype profiles [57]. Ten replicates were carried out, each with the default setting of 100 replicates as burn-in, followed by 100 replicates and a thinning parameter of 10. We specified a boundary that included the full distribution of *V. niloticus* across Africa (electronic supplementary material, S3). The median coordinates across all runs were then plotted in ArcMap 10.1 [58]. Confidence in each CAM estimate was assessed by combining results from all runs and plotting 100 of the 1000 coordinates, weighted by log likelihood. Tighter clustering of the point coordinates indicates a higher confidence in the predicted locality, whereas more disperse point coordinates indicates that the precise locality could not be confidently assigned [57].

We assessed the reliability of the CAM estimates by analysing 30 reference individuals from across the full distribution, 10 from each of the three known genetic lineages within *V. niloticus* [21]. Runs were performed as described above and straight-line distances between actual and estimated localities were calculated. We also examined the geographical clustering versus dispersion pattern of the top 100 point coordinates, weighted according to their log likelihood.

Lastly, to visualize the relationships among reference and introduced individuals, we conducted a principal component analysis (PCA) on raw microsatellite genotypes using the gstudio package [59] in R 3.1 [60].

### 2.4. Ecological niche modelling

We generated ecological niche models (ENMs) for the inferred source population as well as for the full *V. niloticus* species across sub-Saharan Africa. Occurrence points for the source population were limited to reference individuals belonging to the same genetic subclade as the Florida samples. For both ENMs, reference individuals lacking specific locality data were removed from the analysis. In total, nine reference individuals from the African source population and 71 from the full native *V. niloticus* distribution exhibited unique and reliable GPS coordinates and were used in subsequent ENMs (see electronic supplementary material, S1 for GPS coordinates).

We modelled the climatic niche of the *V. niloticus* source population and full species using the elevation and environmental data from the WorldClim database [61] at 2.5 arc-minute resolution. These variables represent the current climate, averaged across the years 1950–2000, and all analyses were carried out using the WGS 1984 projection. The environmental data were reduced to eight variables based on the biology of the organism, visual inspection of the environmental layers and the contribution to model performance based on jackknife replicates performed in Maxent 3.3.3 [62]. The variables selected for the remaining analyses represent annual trends (annual mean temperature, annual precipitation), temperature extremes (temperature seasonality, minimum temperature of the coldest month, mean temperature of the coldest quarter), drought incidence (precipitation of the driest quarter), occurrence of frost (precipitation of the coldest quarter) and elevation.

We generated ENMs using the machine-learning maximum entropy software Maxent 3.3.3 [62]. This approach has been shown to perform well when compared with other methods [63]. Models were built using environmental layers clipped to the African continent with 100 000 background points. The regularization multiplier, which adjusts the specificity of the model to the training points, was varied from 1 to 5 at 0.5 increments and the resulting models were evaluated based on the area under the receiver-operating characteristic curve (AUC) score. Higher AUC scores (closer to 1.0) indicate a greater predictive ability, while scores near 0.5 have poor predictive ability (no better than random). A regularization multiplier of 1.5 was selected for subsequent models.

The final models were evaluated using a fivefold cross-validation method, with AUC scores calculated for each fold along with the omission rate of the test databased on binary predictions with the minimum training presence threshold [64]. We calculated binomial probabilities to assess whether the omission rate was lower than expected compared with a random prediction [65]. ENMs were then projected onto North America without clamping to avoid predicting regions with climatic conditions outside of the model's training range. Suitability maps were then created with the highest level of suitability based on the minimum training presence of the source population so that maps could be directly compared.

To infer the potentially inhabitable regions for *V. niloticus* under future climate projections, we used two global climate models, the Hadley Center for Climate Prediction and Research's model, HadGEM2-ES [66], and the National Center for Atmospheric Research's Community Climate System Model, CCSM4 [67], projected to the years 2050 (average of 2041–2060) and 2070 (average of 2061–2080). We examined projections from two of the four representative concentration pathways (RCPs) used in the Fifth Assessment International Panel on Climate Change (IPCC) report. These emission trajectories range from carbon dioxide concentrations of 421 ppm (RCP 2.6) to 936 ppm (RCP 8.5) by the year 2100 [68]. We selected the second severity level (RCP 4.5, 538 ppm CO_2_) and the most extreme category (RCP 8.5) to examine a range of potential outcomes.

## 3. Results

### 3.1. Phylogenetic and genealogical approaches

**Figure 2.**
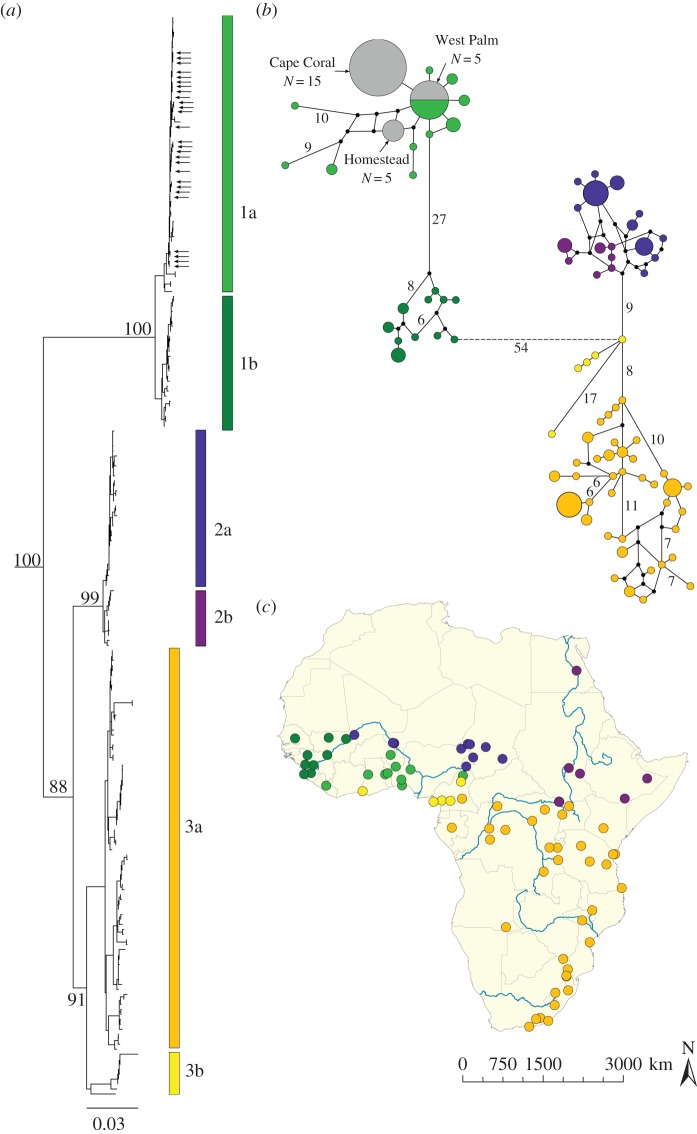
(*a*) Maximum-likelihood tree of *Varanus niloticus*. Outgroup individuals (not shown) include *V. exanthematicus* and *V. albigularis*. The positions of introduced *V. niloticus* sequences are indicated by the arrows. The numbers by major clades represent the bootstrap support (per cent) and the scale bar denotes 0.03 substitutions per site. (*b*) Minimum-spanning network created using the mitochondrial dataset. Circle sizes are proportional to the number of individuals and the colours are consistent with the subclade designations. Mutational steps more than five are indicated and the dashed line represents disjoint subnetworks (less than 95% parsimony threshold). Smaller black circles indicate hypothetical haplotypes. (*c*) Map showing the geographical localities of the reference individuals across Africa.

Our ML analysis, constructed with a concatenated mitochondrial and nuclear dataset, produced geographically structured groups across reference individuals (figure 2*a*; electronic supplementary material, S4) with high bootstrap support. All introduced *V. niloticus* individuals were assigned to subclade 1a, containing reference individuals from a region of coastal West Africa, located from Liberia to Cameroon (figure 2*c*).

The haplotype network, created with the mitochondrial dataset (2094 bp), also assigned all introduced individuals to the 1a subnetwork, indicating that all Florida individuals originated from the southern coastal region of West Africa (figure 2*b*). Each Florida population was represented by a different, fixed mitochondrial haplotype. Introduced individuals from the West Palm Beach population directly shared a haplotype with reference individuals originating from Benin (*N* = 1), Burkina Faso (*N* = 1), Ghana (*N* = 2) and Togo (*N* = 1). There were two mutational differences between the West Palm Beach and Homestead haplotypes, while the Cape Coral and West Palm Beach haplotypes differed from each other by a single base pair.

### 3.2. Frequency-based approaches

The assignment test results were consistent with those obtained from the DNA sequence analyses. All introduced individuals were assigned to the reference population located in southern coastal West Africa, denoted 1a, with probabilities exceeding 0.945 (table 1). All other regions showed lower probabilities, with the more inland West African population ranking second in assignment probabilities.

**Table 1.** Summary of origin estimates for the introduced *Varanus niloticus* individuals in Florida, USA. The clade designations refer to figure 2 and were estimated from mitochondrial and nuclear DNA sequence data. The assignment test results and continuous assignment method (CAM) coordinates were estimated from microsatellite genotypes.

		assignment test (probability)		CAM coordinates	
individual ID	clade	first	second	latitude	longitude
Cape Coral 6832	1a	1a (0.988)	1b (0.522)	13.4714	−10.8562
Cape Coral 6915	1a	1a (0.974)	1b (0.422)	11.1075	−7.7224
Cape Coral 6918	1a	1a (0.983)	1b (0.418)	7.7639	−6.2267
Cape Coral 6924	1a	1a (0.989)	1b (0.485)	13.1684	−8.5857
Cape Coral 6926	1a	1a (0.974)	1b (0.422)	10.6039	−6.341
Cape Coral 6938	1a	1a (0.999)	1b (0.663)	9.1802	−6.2612
Cape Coral 6960	1a	1a (0.986)	1b (0.500)	10.2182	−4.6976
Cape Coral 6963	1a	1a (0.994)	1b (0.508)	8.6538	−3.0581
Cape Coral 6966	1a	1a (0.972)	1b (0.470)	10.0618	−12.1069
Cape Coral 6971	1a	1a (0.968)	1b (0.373)	10.3063	−4.2984
Cape Coral 7000	1a	1a (0.994)	1b (0.508)	8.1689	−0.7365
Cape Coral 7001	1a	1a (0.994)	1b (0.508)	6.8945	−1.9764
Cape Coral 7011	1a	1a (0.979)	1b (0.490)	11.0955	−13.9507
Cape Coral 7017	1a	1a (0.977)	1b (0.500)	13.387	−7.9643
Cape Coral 7018	1a	1a (0.974)	1b (0.435)	8.5988	−11.1605
Homestead 1	1a	1a (0.999)	1b (0.622)	13.4714	−10.8562
Homestead 2	1a	1a (0.990)	1b (0.676)	9.1257	−1.233
Homestead 3	1a	1a (0.999)	1b (0.512)	8.7121	−7.2019
Homestead 4	1a	1a (0.999)	1b (0.636)	8.0381	−0.0763
Homestead 5	1a	1a (0.999)	1b (0.523)	9.4268	−1.9467
West Palm 1	1a	1a (0.999)	1b (0.499)	9.1805	−5.8981
West Palm 2	1a	1a (1.000)	1b (0.601)	13.1922	−6.0551
West Palm 3	1a	1a (0.999)	1b (0.499)	10.24	−1.9004
West Palm 4	1a	1a (0.999)	1b (0.472)	12.5839	−1.4289
West Palm 5	1a	1a (0.945)	1b (0.083)	10.2728	−3.109

The CAM analysis implemented in SCAT showed varying levels of accuracy and confidence in assigning reference individuals to their locality of origin. Overall, median locality estimates were most accurate for reference individuals in the western clade (1a and 1b), assigning the geographical origin to within an average of 520 km from the actual origin (range 69–1082 km). Reference individuals from the northern (2a and 2b) and southern (3a and 3b) clades produced lower levels of accuracy, with straight-line distances between estimated and actual localities averaging 845 km (range 119–2318 km) and 984 km (range 236–1974 km), respectively. Similarly, confidence in the estimated origin was generally higher for the western clade, which exhibited tighter geographical clustering of point coordinates (e.g. electronic supplementary material, S5). These estimates from reference individuals provide a general idea of the level and accuracy and precision we can expect in identifying the origin of the introduced individuals with the CAM.

**Figure 3.**
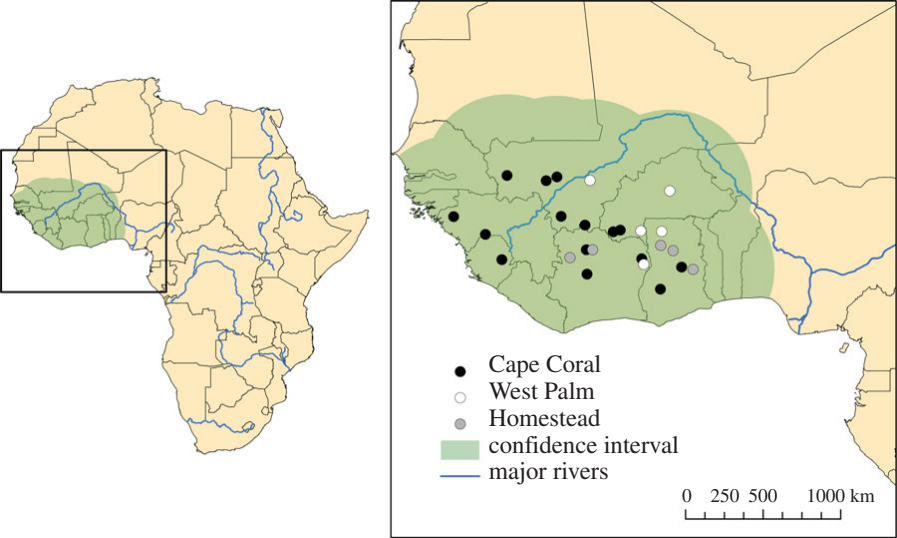
Continuous assignment method (CAM) results showing the median point coordinates of all introduced *Varanus niloticus* individuals, from the Cape Coral, West Palm Beach and Homestead localities in Florida. The confidence interval around each coordinate (520 km) is based on the average accuracy for reference individuals within the western clade.

Median point coordinate estimates for all introduced *V. niloticus* individuals were located within West Africa (figure 3 and table 1). Unlike previous analyses, the estimated origins span the full region of the western clade, present in the coastal region as well as further inland and west. However, a majority of the Florida individuals (19/25) were estimated to originate in the coastal region, southeast of the Niger River.

**Figure 4.**
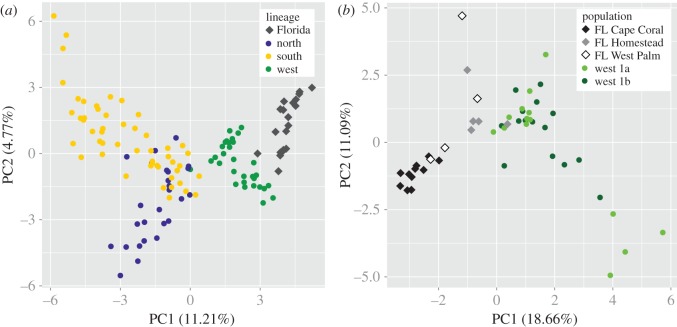
Principal component analysis (PCA) of microsatellite genotype data from native and introduced *Varanus niloticus* individuals showing all major genetic lineages (*a*) and western subclades (*b*).

The PCA plot showing major *V. niloticus* lineages clearly grouped all introduced individuals within the western cluster (figure 4). However, examining the western and Florida populations alone could not further distinguish the 1a or 1b subclade as the source (figure 4). Furthermore, the Cape Coral population, residing in Florida for a greater time period than the other introduced populations (first observed in 1990, compared with 2000 and 2004 for the West Palm Beach and Homestead populations, respectively), showed greater overall separation from the reference individuals and formed a tighter cluster. Conversely, the Homestead and West Palm Beach individuals were more diffuse and grouped closely with the reference genotypes.

### 3.3. Ecological niche modelling

**Figure 5.**
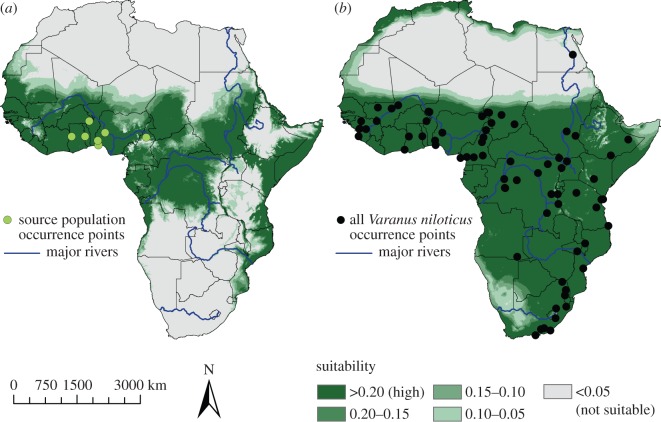
Ecological niche model (ENM) of the *Varanus niloticus* source population (*a*) and full species distribution (*b*) across Africa. The occurrence points are shown and the highest level of suitability is based on the minimum training presence.

From the results of the DNA sequence analyses, supplemented by the assignment tests, we determined the source population to be the subclade denoted 1a, found throughout coastal West Africa. The ENM constructed from this West African source population (nine occurrence points) produced an average test AUC of 0.924 (0.824–0.97), and an average omission error rate from the binomial test of 0 for all but onefold (*p* = 0.028–0.281) and 0.5 for the remaining fold (*p* = 0.148). For the full *V. niloticus* distribution (71 occurrence points), the ENM produced and average test AUC of 0.770 (0.683–0.830), and an average omission error rate of 0.029 (*p* = 0.007–0.084). Projecting the source population ENM onto the African continent showed that coastal West Africa was predicted to be highly suitable (greater than 0.2 based on the minimum training presence threshold) and produced a more restricted range than the full *V. niloticus* ENM (figure 5).

**Figure 6.**
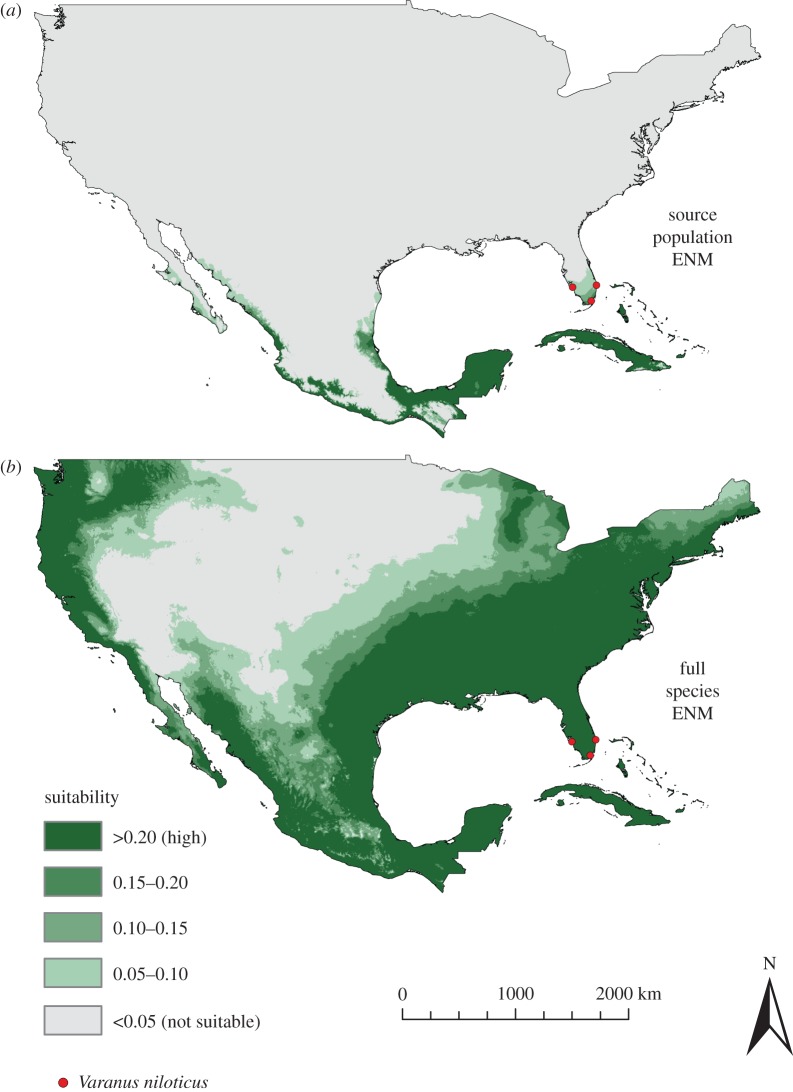
Ecological niche model (ENM) of the *Varanus niloticus* source population (*a*) and full species distribution (*b*) projected onto the current climate conditions of North America. The location of the introduced populations is shown and the highest level of suitability is based on the minimum training presence.

Projecting the West African source population ENM onto North America under the current climate showed that suitable climatic conditions were predicted to occur in the southern portion of Florida (figure 6). This suitable region contained all three introduced populations, with the Cape Coral population occurring in the northernmost extent of the estimated range. Both the Cape Coral and the West Palm Beach populations occurred in regions of low climatic suitability, while the Homestead population was found in highly suitable conditions. In other areas, highly suitable climates were found in Cuba, Caribbean islands and coastal areas of Mexico, although *V. niloticus* occurrences have not yet been documented in these regions.

In contrast with the localized distribution predicted from the West African source population, the ENM based on conditions throughout the full sub-Saharan range of *V. niloticus* predicted suitable climates across a large expanse of North America. Suitable climatic conditions occurred throughout the eastern United States, a majority of Mexico and portions of the West Coast, as well as the Caribbean islands and Cuba.

**Figure 7.**
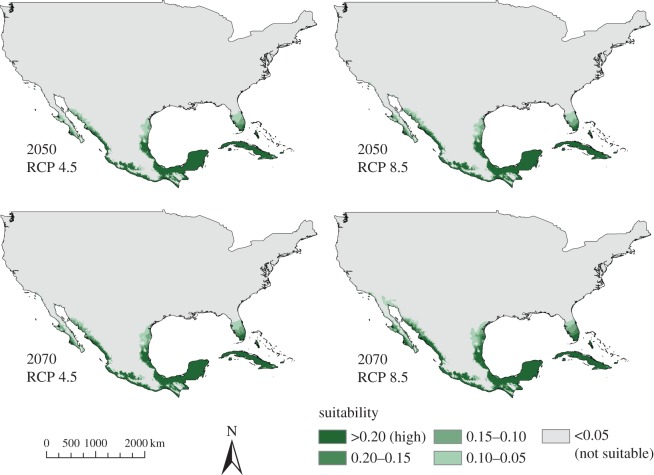
Ecological niche model (ENM) based on the *Varanus niloticus* source population showing future climate projections with the Hadley Center global climate model (HadGEM2-ES) for the years 2050 and 2070. Representative concentration pathways (RCPs) of 4.5 (low–moderate climate scenario) and 8.5 (most extreme scenario) are shown.

**Figure 8.**
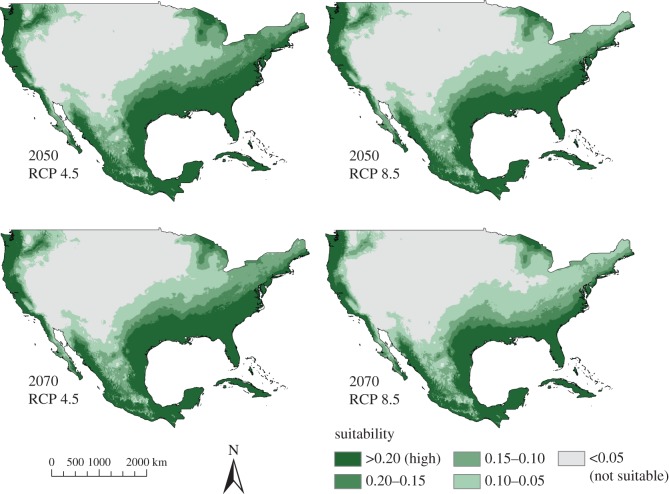
Ecological niche model (ENM) based on the full *Varanus niloticus* distribution showing future climate projections with the Hadley Center global climate model (HadGEM2-ES) for the years 2050 and 2070. Representative concentration pathways (RCPs) of 4.5 (low–moderate climate scenario) and 8.5 (most extreme scenario) are shown.

For both ENMs, the predicted suitability under future climate projections was consistent across global climate models (see electronic supplementary material, S6 and S7 for CCSM4 model). For these future projections, areas predicted to be suitable for the West African source population extended farther north than under current climatic conditions; however, for the United States, the potentially inhabitable areas were confined to Florida, as well as small portions of Texas and California (figure 7). For the West African ENM, the more extreme global climate change scenario (RCP 8.5) predicted a larger area of suitable climate than the moderate scenario (RCP 4.5). By contrast, the area predicted to be suitable for *V. niloticus* as a whole became more restricted under future climate projections, with the more extreme scenarios (RCP 8.5) showing smaller predicted ranges (figure 8).

## 4. Discussion

### 4.1. Geographical origin and the pet trade

Our molecular analyses based on DNA sequence data and microsatellite genotypes identified the source population of all three Florida *V. niloticus* populations to be the lineage inhabiting coastal West Africa. Reference individuals within this subclade range from Liberia to Cameroon, with the northernmost occurrence in Burkina Faso. The DNA sequence data produced a greater degree of genetic partitioning among reference populations and were able to assign Florida individuals more precisely than the microsatellite data. Introduced *V. niloticus* individuals were recovered within the coastal West African subclade, visualized in the ML tree and haplotype network (figure 2). The results from our microsatellite analyses assigned all introduced individuals within the western reference lineage; however, differentiating the two western subclades proved more ambiguous.

These results are consistent with import and export data of live *V. niloticus* for the pet trade. In the 15-year period between 1999 and 2013, the majority of live *V. niloticus* legally imported into the United States originated from Togo and Benin, with reports totalling 66 500 and 45 000 individuals, respectively, and averaging 3000–4500 individuals imported from each country annually [69]. Although the United States frequently imports live *V. niloticus* from other countries, including Tanzania (590 individuals per year average), Ghana (264 individuals per year average) and Niger (92 individuals per year average), these numbers pale in comparison with imports from Togo and Benin [69]. This prolonged level of concentrated exploitation is cause for concern, beyond that of potential non-native introductions. Considering the relatively small geographical area contained by Togo and Benin, exporting over 7000 individuals annually could have detrimental effects on the native *V. niloticus* populations. Furthermore, populations of *V. niloticus* in West Africa are also intensely harvested for the leather industry, with the top exporting country, Mali, reporting an annual average of approximately 47 000 skins (http://trade.cites.org). While *V. niloticus* is managed as a single taxonomic unit by the Convention on International Trade in Endangered Species (CITES, Appendix II) and considered a low conservation concern due to its widespread distribution [70], Dowell *et al.* [21] identified the populations inhabiting West Africa to be genetically distinct from those throughout the rest of the continent. Additionally, previous genetic assessments found reduced variation within the western population compared with neighbouring *V. niloticus* populations [40]. Therefore, the cryptic *V. niloticus* lineage in West Africa could represent a greater conservation concern than previously thought.

Our results suggest that the introduced *V. niloticus* populations inhabiting Cape Coral, West Palm Beach and Homestead resulted from three separate introduction events. This inference was based on differing mitochondrial haplotypes among each of the introduced populations. We found that each Florida population shared a single mitochondrial haplotype, indicative of low genetic diversity; however, independent introductions and subsequent admixture could increase the genetic variation, and possibly the invasive potential, of introduced species [71,72]. These results, in addition to the *V. niloticus* sightings in other parts of Florida [16], call for increased preventative measures as well as containment of gene flow among existing populations.

### 4.2. Inhabitable regions and conservation implications

Based on the climatic conditions of the West African source population, the regions predicted to have suitable climates for the introduced *V. niloticus* populations in the United States were confined to southern Florida, even in future climate projections. These potentially inhabitable regions represent the most specific outcome and are based on the assumption that the West African lineage exhibits local adaptation. Under this assumption, individuals belonging to the western lineage, including the Florida individuals, would have a fitness advantage in environmental conditions most similar to those in West Africa, and reduced fitness levels in other environments. Because reduced gene flow among populations in heterogeneous environments can facilitate local adaptation [22,23], this is a plausible scenario for the *V. niloticus* lineages. In this outcome, all three introduced populations reside in regions predicted to be suitable under current climate conditions; however, the Cape Coral population is near the northernmost extent of the predicted distribution. Although confirmed sightings of *V. niloticus* have occurred outside of this predicted range [16], there is no evidence of reproducing individuals in other localities [16], possibly suggesting reduced fitness at higher latitudes.

Without information regarding the relative fitness of *V. niloticus* genotypes in varying environmental conditions, it is necessary to examine alternative outcomes. The contrasting scenario shows the potentially inhabitable regions based on climate data throughout the full native *V. niloticus* range spanning sub-Saharan Africa. This prediction is based on the assumption that members of the western lineage, as well as those in Florida, do not exhibit local adaptation, but instead show similar fitness levels in all environmental conditions throughout the full native range of the species. Under this assumption, introduced *V. niloticus* individuals would be able to tolerate climatic conditions throughout a large expanse of the United States and Mexico, as well as Cuba and the Caribbean islands. Because some populations of *V. niloticus* are known to hibernate in dry, cool periods [19,73], introduced individuals could potentially withstand temperate winter conditions. Although dispersal distance would likely prevent the introduced populations in Florida from spreading throughout the full range of suitable climates in North America, this scenario predicts that future introductions of *V. niloticus* could potentially lead to additional populations in regions other than Florida. It is important to note, however, that both models were created based solely on climate and elevation data. Therefore, more specific habitat conditions, including permanent bodies of water and appropriate substrate for egg laying [17], could also affect whether *V. niloticus* is able to become established in a particular region. Future studies assessing the degree of local adaptation among native *V. niloticus* lineages could help distinguish between these two contrasting scenarios.

Understanding the geographical origin of the introduced *V. niloticus* populations can aid in focusing management strategies and eradication efforts. Even under the most conservative scenario, our analyses showed that the southern regions of Florida are highly suitable for *V. niloticus*. Therefore, the immediate management focus should target the southernmost regions, and wildlife officials should act quickly when new *V. niloticus* individuals or populations are observed in these areas. Because *V. niloticus* individuals occupy large home ranges and are capable of dispersing large distances [17], sensitive areas including the Everglades National Park and Big Cypress National Preserve could potentially be in danger of invasion. This information heightens our concern for the negative impact that *V. niloticus* may have on native wildlife species in these regions, including ground-nesting birds, aquatic turtles and other vulnerable species, such as the American crocodile and the gopher tortoise [15]. Although Florida has taken steps to limit the number of *V. niloticus* imports [16], other southern states should consider similar precautionary measures to prevent the occurrence of future *V. niloticus* populations.

## 5. Conclusion

Our study illustrates the importance of developing range-wide genetic assessments and their utility in conservation. This library of genetic variation across a species' distribution can serve as a database to source unknown individuals, with applications to invasion biology [33,71,74] as well as to wildlife trafficking, determining the origin of illegally shipped wildlife products [57,75–77]. Using the phylogeographic patterns of *V. niloticus* across Africa, we determined the source of all three introduced populations to be the cryptic West African evolutionary lineage. Furthermore, by identifying the geographical origin of the introduced *V. niloticus* populations, we have established a baseline from which future studies can expand upon. With this information, subsequent investigations can make direct comparisons between introduced and native populations, comparing diet, morphology, gene expression, etc. to examine if and how *V. niloticus* is adapting to the non-native habitat.

## Supplementary Material

Supplemental file 1 Table of locality and collector information for all Varanus niloticus reference individuals and introduced populations

## Supplementary Material

Supplemental file 2 Spreadsheet of microsatellite genotypes for reference and Florida Varanus niloticus individuals

## Supplementary Material

Supplemental file 3 GPS coordinates in the boundary file used for the continuous assignment method (CAM) in SCAT

## Supplementary Material

Supplemental file 4 Maximum likelihood tree in Newick format

## Supplementary Material

Supplemental file 5 Continuous Assignment Method (CAM) results showing select Varanus niloticus reference individuals from each subclade, delineated by differing colors. Stars represent the actual locality and crosses mark the median estimated locality, averaged across 10 runs. The surrounding point coordinates show the confidence in the estimated locality, with geographically clustered points indicating high confidence and more diffuse points representing low confidence. These point coordinates were compiled across all runs and 100 were selected based on the highest log likelihood scores

## Supplementary Material

Supplemental file 6 Ecological niche model (ENM) of the Varanus niloticus source population showing future climate projections with the National Center for Atmospheric Research's Community Climate System Model (CCSM4) for the years 2050 and 2070. Representative Concentration Pathways (RCPs) of 4.5 (low-moderate climate scenario) and 8.5 (most extreme scenario) are shown

## Supplementary Material

Supplemental file 7 Ecological niche model (ENM) of the full Varanus niloticus distribution showing future climate projections with the National Center for Atmospheric Research's Community Climate System Model (CCSM4) for the years 2050 and 2070. Representative Concentration Pathways (RCPs) of 4.5 (low-moderate climate scenario) and 8.5 (most extreme scenario) are shown

## Supplementary Material

Supplemental Material Legends

## Data accessibility

DNA sequences: GenBank accessions KT894381–KT894551. The remaining datasets supporting this article have been uploaded as part of the electronic supplementary material.
