# Supplementary material for: Combining genetic and distributional approaches to sourcing introduced species: a case study on the Nile monitor (Varanus niloticus) in Florida
Source: R Soc Open Sci. 2016 Apr 20;3(4):150619. doi: 10.1098/rsos.150619 (PMC4852627; doi:10.1098/rsos.150619)
Supplement: Supplemental file 1 Table of locality and collector information for all Varanus niloticus reference individuals and introduced populations [file rsos150619supp1.docx]

**Supplemental file 1** Table of contemporary samples and museum specimens of *Varanus niloticus* utilized in this study. The subclade designation refers to figure 2 and the specimen ID refers to the field number (contemporary) or museum accession number (archival specimens). Museum acronyms: AMNH–American Museum of Natural History (New York, NY, USA), CAS–California Academy of Sciences (San Francisco, CA, USA), FLMNH–Florida Museum of Natural History (Gainesville, FL, USA), MCZ–Museum of Comparative Zoology, Harvard (Cambridge, MA, USA), MNHN-RA– Muséum national d’Histoire naturelle (Paris, France), MVZ–Museum of Vertebrate Zoology (Berkeley, CA, USA), NHM–Natural History Museum (London, UK), PEM–Port Elizabeth Museum, Bayworld (Port Elizabeth, South Africa), UMMZ–University of Michigan Museum of Zoology (Ann Arbor, MI, USA), USNM–National Museum of Natural History, Smithsonian Institution (Washington, DC, USA) , UTEP – University of Texas, El Paso (El Paso, TX, USA), UWBM–Burke Museum of Natural History and Culture (Seattle, WA, USA), SANBI–South Africa National Biodiversity Institute (Silverton, South Africa).

| Subclade | Country | Locality | Museum/Collector | Specimen ID | Coordinates | |
| --- | --- | --- | --- | --- | --- | --- |
|  |  |  |  |  | Latitude | Longitude |
| West-1a | Cameroon | Gamba | I. Ineich | Ineich80 | 8.1* | 13.602* |
| West-1a | Nigeria | Lagos | NHM | 1893.1.11.10**–**11 | 6.4541001* | 3.38943* |
| West-1a | Nigeria | Jebba | NHM | 1899.8.23.8–9 | 9.1164799* | 4.82164* |
| West-1a | Nigeria | Igbo-Ora | USNM | 194862 | 7.4367199* | 3.28864* |
| West-1a | Benin | Country Only | MNHN-RA | 1894.0259 | N/A | N/A |
| West-1a | Benin | Country Only | MNHN-RA | 1894.0260 | N/A | N/A |
| West-1a | Togo | Country Only | FLMNH | 56590 | N/A | N/A |
| West-1a | Togo | Country Only | FLMNH | 60998 | N/A | N/A |
| West-1a | Togo | Country Only | FLMNH | 61009 | N/A | N/A |
| West-1a | Togo | Country Only | FLMNH | 61037 | N/A | N/A |
| West-1a | Togo | Country Only | FLMNH | 61468 | N/A | N/A |
| West-1a | Togo/Liberia | Country Only | FLMNH | 63940 | N/A | N/A |
| West-1a | Ghana | Volta Region | MVZ | 245359 | 8.32511* | 0.55622* |
| West-1a | Ghana | Ahato Region | UWBM | 6058 | 8.29326* | -2.28347* |
| West-1a | Ghana | Brong-Ahato Region | UWBM | 6059 | 8.29635* | -2.28542* |
| West-1a | Ghana | Brong-Ahato Region | UWBM | 6060 | 8.29453* | -2.2827* |
| West-1a | Burkina Faso | Arly | USNM | 223945** | 11.5832996* | 1.46667* |
| West-1a | Liberia | Country Only | FLMNH | 61065 | N/A | N/A |
| West-1b | Sierra Leone | Country Only | PEM | R19410 | N/A | N/A |
| West-1b | Sierra Leone | Newton Colony | MCZ | 53577 | 8.335169 | -13.008333 |
| West-1b | Sierra Leone | Mussia | MCZ | 53578 | 9.864203 | -11.429656 |
| West-1b | Guinea | Killissi | I. Ineich | Ineich550 | 9.95 | -12.816667 |
| West-1b | Guinea | Djakka | I. Ineich | Ineich554 | 11.54181 | -12.4517 |
| West-1b | Guinea | Siguiri | I. Ineich | Ineich565 | 11.566778 | -9.138417 |
| West-1b | Senegal | Country Only | NHM | 1920.1.20.66 | N/A | N/A |
| West-1b | West Mali | Flabougou | V. de Buffrénil | FLA22 | 14.47361111 | -8.892777778 |
| West-1b | West Mali | Flabougou | V. de Buffrénil | FLA23 | 14.47361111 | -8.892777778 |
| West-1b | West Mali | Flabougou | V. de Buffrénil | FLA24 | 14.47361111 | -8.892777778 |
| West-1b | West Mali | Flabougou | V. de Buffrénil | FLA25 | 14.47361111 | -8.892777778 |
| West-1b | West Mali | Niono | V. de Buffrénil | NIONO4 | 14.25333333 | -5.984722222 |
| West-1b | West Mali | Niono | V. de Buffrénil | NIONO5 | 14.25333333 | -5.984722222 |
| West-1b | West Mali | Niono | V. de Buffrénil | NIONO6 | 14.25333333 | -5.984722222 |
| West-1b | West Mali | Niono | V. de Buffrénil | NIONO7 | 14.25333333 | -5.984722222 |
| North-2a | East Mali | Mopti | V. de Buffrénil | MOP96_20 | 14.94333333 | -4.572222222 |
| North-2a | East Mali | Mopti | V. de Buffrénil | MOP96_66 | 14.94333333 | -4.572222222 |
| North-2a | East Mali | Mopti | V. de Buffrénil | MOP96_78 | 14.94333333 | -4.572222222 |
| North-2a | East Mali | Mopti | V. de Buffrénil | MOP96_79 | 14.94333333 | -4.572222222 |
| North-2a | Niger | Niamey | V. de Buffrénil | NIA34 | 13.51694444 | 2.1 |
| North-2a | Niger | Niamey | V. de Buffrénil | NIA35 | 13.51694444 | 2.1 |
| North-2a | Niger | Niamey | MVZ | 238936 | 13.616667 | 1.866667 |
| North-2a | Niger | Diffa | V. de Buffrénil | DIF47 | 12.61666667 | 13.31666667 |
| North-2a | Niger | Diffa | V. de Buffrénil | DIF48 | 12.61666667 | 13.31666667 |
| North-2a | Niger | Diffa | V. de Buffrénil | DIF51 | 12.61666667 | 13.31666667 |
| North-2a | Niger | Diffa | V. de Buffrénil | DIF53 | 12.61666667 | 13.31666667 |
| North-2a | Chad | Dassoulom | I. Ineich | Ineich603 | 13.351994 | 14.791903 |
| North-2a | Chad | Am N’Guitey | V. de Buffrénil | AMNG6 | 10.90416667 | 20.22694444 |
| North-2a | Chad | Am N’Guitey | V. de Buffrénil | AMNG7 | 10.90416667 | 20.22694444 |
| North-2a | Chad | Abba Liman | V. de Buffrénil | ABLIM1 | 11.15916667 | 15.32527778 |
| North-2a | Chad | Abba Liman | V. de Buffrénil | ABLIM2 | 11.15916667 | 15.32527778 |
| North-2a | Chad | Lake Chad | V. de Buffrénil | LCDJA151 | 13.41666667 | 14.43333333 |
| North-2a | Chad | Lake Chad | V. de Buffrénil | LCDJA152 | 13.41666667 | 14.43333333 |
| North-2a | Chad | Lake Fitri | V. de Buffrénil | LFKOUR239 | 12.88333333 | 17.43333333 |
| North-2a | Chad | Lake Fitri | V. de Buffrénil | LFKOUR243 | 12.88333333 | 17.43333333 |
| North-2a | Chad | Lake Lere | V. de Buffrénil | LLKD260 | 9.636666667 | 14.16111111 |
| North-2a | Chad | Lake Lere | V. de Buffrénil | LLKD261 | 9.636666667 | 14.16111111 |
| North-2b | Egypt | Luxor | NHM | 1897.10.28.224 | 25.695694 | 32.6473999 |
| North-2b | Egypt | Luxor | NHM | 1906.7.25.3 | 25.695694 | 32.6473999 |
| North-2b | Sudan | White Nile | NHM | 6526 | 9.3808002 | 31.3418999 |
| North-2b | Ethiopia | Awharra, Mullka | NHM | 1902.12.13.38 | 4.2606578 | 40.7323723 |
| North-2b | Ethiopia | Barro River (Sobat) | NHM | 1905.10.16.5 | 8.4361115 | 33.2186127 |
| North-2b | Ethiopia | Ogaden Region | AMNH | 19445 | 7.6263223 | 44.4752502 |
| North-2b | Lang Chapin | Unknown | AMNH | 74604 | N/A | N/A |
| North-2b | DRC | Uele | AMNH | 10512 | 3.7099299 | 29.7139301 |
| North-2b | DRC | Uele | AMNH | 10517 | 3.7099299 | 29.7139301 |
| North-2b | DRC | Uele | AMNH | 10519 | 3.7099299 | 29.7139301 |
| South-3a | DRC | Ituri | AMNH | 10500 | 1.5666699 | 30.25 |
| South-3a | DRC | Ituri | AMNH | 10501 | 2.3927 | 27.3119602 |
| South-3a | DRC | Ituri | AMNH | 10502 | 2.3927 | 27.3119602 |
| South-3a | DRC | Stanleyville | AMNH | 10085 | 0.51654 | 25.2010002 |
| South-3a | DRC | Tandala | USNM | 216247 | 2.9671199 | 19.3633995 |
| South-3a | DRC | Kyolo, Katanga | E. Greenbaum | UTEP21180 | -8.02 | 27.1165 |
| South-3a | DRC | Kaputo, South Kivu | E. Greenbaum | UTEP21182 | -3.954 | 28.0869 |
| South-3a | DRC | North of Kibili | E. Greenbaum | UTEP21183 | -3.954 | 28.0869 |
| South-3a | DRC | Bokaka, Lake Tumba | E. Greenbaum | UTEP21184 | -0.782133 | 18.027525 |
| South-3a | DRC | North of Kutu | E. Greenbaum | UTEP21185 | -2.639847 | 18.148431 |
| South-3a | DRC | Lac Mai-Ndomb | E. Greenbaum | UTEP21186 | -2.69925 | 18.231592 |
| South-3a | DRC | Equateur: Efofa | E. Greenbaum | UTEP21187 | -1.00322 | 20.70763 |
| South-3a | Uganda | Rhino Camp | USNM | 42260 | 2.97137 | 31.3940392 |
| South-3a | Kenya | Fort Hall | NHM | 1906.8.25.1 | -0.72312 | 37.1612015 |
| South-3a | Tanzania | Shinyanga | USNM | 72028 | -3.6777301 | 33.4034004 |
| South-3a | Tanzania | Siga Caves | MCZ | 47377 | -5.066667 | 39.1 |
| South-3a | Tanzania | Magrotto Mtn. | MCZ | 47378 | -5.116667 | 38.75 |
| South-3a | Tanzania | Country Only | MNHN-RA | 5610 | N/A | N/A |
| South-3a | Tanzania | Morogoro | UMMZ | 61412 | -6.7860599 | 37.6651001 |
| South-3a | Tanzania | Tanganyika | NHM | 1927.5.27.10 | -6.0803699 | 29.5619831 |
| South-3a | Tanzania | Tanganyika | NHM | 1927.9.27.11 | -6.0803699 | 29.5619831 |
| South-3a | Burundi | Rumonge | E. Greenbaum | UTEP21181 | -3.98023 | 29.44258 |
| South-3a | Angola | Cunga | NHM | 1904.5.2.27 | -17.2999992 | 20.7999992 |
| South-3a | Malawi | Fort Johnston | NHM | 1926.5.8.14 | -14.4787598 | 35.2725983 |
| South-3a | Mozambique | Tete | R. Fergusson | MZ01 | -16.1583996 | 33.5880013 |
| South-3a | Mozambique | Tete | R. Fergusson | MZ02 | -16.1583996 | 33.5880013 |
| South-3a | Mozambique | Tete | R. Fergusson | MZ03 | -16.1583996 | 33.5880013 |
| South-3a | Mozambique | Tete | R. Fergusson | MZ04 | -16.1583996 | 33.5880013 |
| South-3a | Mozambique | Tete | R. Fergusson | MZ05 | -16.1583996 | 33.5880013 |
| South-3a | Mozambique | Tete | R. Fergusson | MZ06 | -16.1583996 | 33.5880013 |
| South-3a | Mozambique | Tete | R. Fergusson | MZ07 | -16.1583996 | 33.5880013 |
| South-3a | Mozambique | Nhica do Rovuma | I. Ineich | Ineich371 | -10.7325001 | 40.2150002 |
| South-3a | Mozambique | Beira | NHM | 1907.4.29.32 | -19.835361 | 34.854469 |
| South-3a | South Africa | North of Humansdorp | SANBI | 410 | -33.994444 | 24.703333 |
| South-3a | South Africa | Country Only | SANBI | 3483 | N/A | N/A |
| South-3a | South Africa | Wilgepark | SANBI | 3697 | -28.289167 | 29.089167 |
| South-3a | South Africa | Edendale | SANBI | 5308 | -30.387778 | 28.971667 |
| South-3a | South Africa | Phalaborwa | SANBI | 5456 | -24.385556 | 31.140278 |
| South-3a | South Africa | Limpopo | MVZ | 267341 | -22.631778 | 30.399361 |
| South-3a | South Africa | Cookhouse | PEM | WC10-180 | -32.714528 | 25.861861 |
| South-3a | South Africa | Brown’s Vale | PEM | WC10-194 | -32.520389 | 26.536139 |
| South-3a | South Africa | Nelspruit | L. La Grange | SA01 | -25.546048 | 30.955292 |
| South-3a | South Africa | Nelspruit | L. La Grange | SA02 | -25.461997 | 30.974866 |
| South-3a | South Africa | Nelspruit | L. La Grange | SA03 | -25.461997 | 30.974866 |
| South-3a | South Africa | Ngoye Hills, Zuzuland | NHM | 1905.3.7.102 | -27.89748 | 31.2474098 |
| South-3a | South Africa | Ngoye Hills, Zuzuland | NHM | 1905.3.7.103 | -27.89748 | 31.2474098 |
| South-3a | South Africa | East London | MNHN-RA | 1896.0374 | -33.0154419 | 27.9040298 |
| South-3a | South Africa | East London | MNHN-RA | 1896. 0375 | -33.0154419 | 27.9040298 |
| South-3b | Ivory Coast | Elima | MNHN-RA | 6452 | 5.4913454 | -3.2038913 |
| South-3b | Eq. Guinea | Bioko | CAS | 207622 | 3.710917 | 8.666639 |
| South-3b | Cameroon | Metet | AMNH | 36473 | 7.0833302 | 13.2833004 |
| South-3b | Cameroon | Yaounde | I. Ineich | Ineich81 | 3.8729999 | 11.5222998 |
| South-3b | Cameroon | Yaounde | I. Ineich | 765 | 3.8729999 | 11.5222998 |
| South-3b | Cameroon | West of Edea | Pending | DMP519 | 3.935833 | 10.056667 |
| South-3b | Cameroon | Doume | MNHN-RA | 5281 | 4.23524 | 13.4534101 |
| South-3b | Cameroon | Doume | MNHN-RA | 2007.2424 | 4.23524 | 13.4534101 |
| South-3b | Gabon | Country Only | MNHN-RA | 8265 | N/A | N/A |
| South-3b | Gabon | Country Only | MNHN-RA | 2007.2425 | N/A | N/A |
| West-1a | USA-Florida | Cape Coral | T. Campbell | CC 6832 | N/A | N/A |
| West-1a | USA-Florida | Cape Coral | T. Campbell | CC 6915 | N/A | N/A |
| West-1a | USA-Florida | Cape Coral | T. Campbell | CC 6918 | N/A | N/A |
| West-1a | USA-Florida | Cape Coral | T. Campbell | CC 6924 | N/A | N/A |
| West-1a | USA-Florida | Cape Coral | T. Campbell | CC 6926 | N/A | N/A |
| West-1a | USA-Florida | Cape Coral | T. Campbell | CC 6938 | N/A | N/A |
| West-1a | USA-Florida | Cape Coral | T. Campbell | CC 6960 | N/A | N/A |
| West-1a | USA-Florida | Cape Coral | T. Campbell | CC 6963 | N/A | N/A |
| West-1a | USA-Florida | Cape Coral | T. Campbell | CC 6966 | N/A | N/A |
| West-1a | USA-Florida | Cape Coral | T. Campbell | CC 6971 | N/A | N/A |
| West-1a | USA-Florida | Cape Coral | T. Campbell | CC 7000 | N/A | N/A |
| West-1a | USA-Florida | Cape Coral | T. Campbell | CC 7001 | N/A | N/A |
| West-1a | USA-Florida | Cape Coral | T. Campbell | CC 7011 | N/A | N/A |
| West-1a | USA-Florida | Cape Coral | T. Campbell | CC 7017 | N/A | N/A |
| West-1a | USA-Florida | Cape Coral | T. Campbell | CC 7018 | N/A | N/A |
| West-1a | USA-Florida | Homestead |  | H1 | N/A | N/A |
| West-1a | USA-Florida | Homestead |  | H2 | N/A | N/A |
| West-1a | USA-Florida | Homestead |  | H3 | N/A | N/A |
| West-1a | USA-Florida | Homestead |  | H4 | N/A | N/A |
| West-1a | USA-Florida | Homestead |  | H5 | N/A | N/A |
| West-1a | USA-Florida | West Palm Beach |  | WP1 | N/A | N/A |
| West-1a | USA-Florida | West Palm Beach |  | WP2 | N/A | N/A |
| West-1a | USA-Florida | West Palm Beach |  | WP3 | N/A | N/A |
| West-1a | USA-Florida | West Palm Beach |  | WP4 | N/A | N/A |
| West-1a | USA-Florida | West Palm Beach |  | WP5 | N/A | N/A |

*Coordinates used to generate model of West African source population

**Originally mis-identified as *V. exanthematicus*
