# Supplementary figures and images for: Combining genetic and distributional approaches to sourcing introduced species: a case study on the Nile monitor (Varanus niloticus) in Florida
Source: R Soc Open Sci. 2016 Apr 20;3(4):150619. doi: 10.1098/rsos.150619 (PMC4852627; doi:10.1098/rsos.150619)

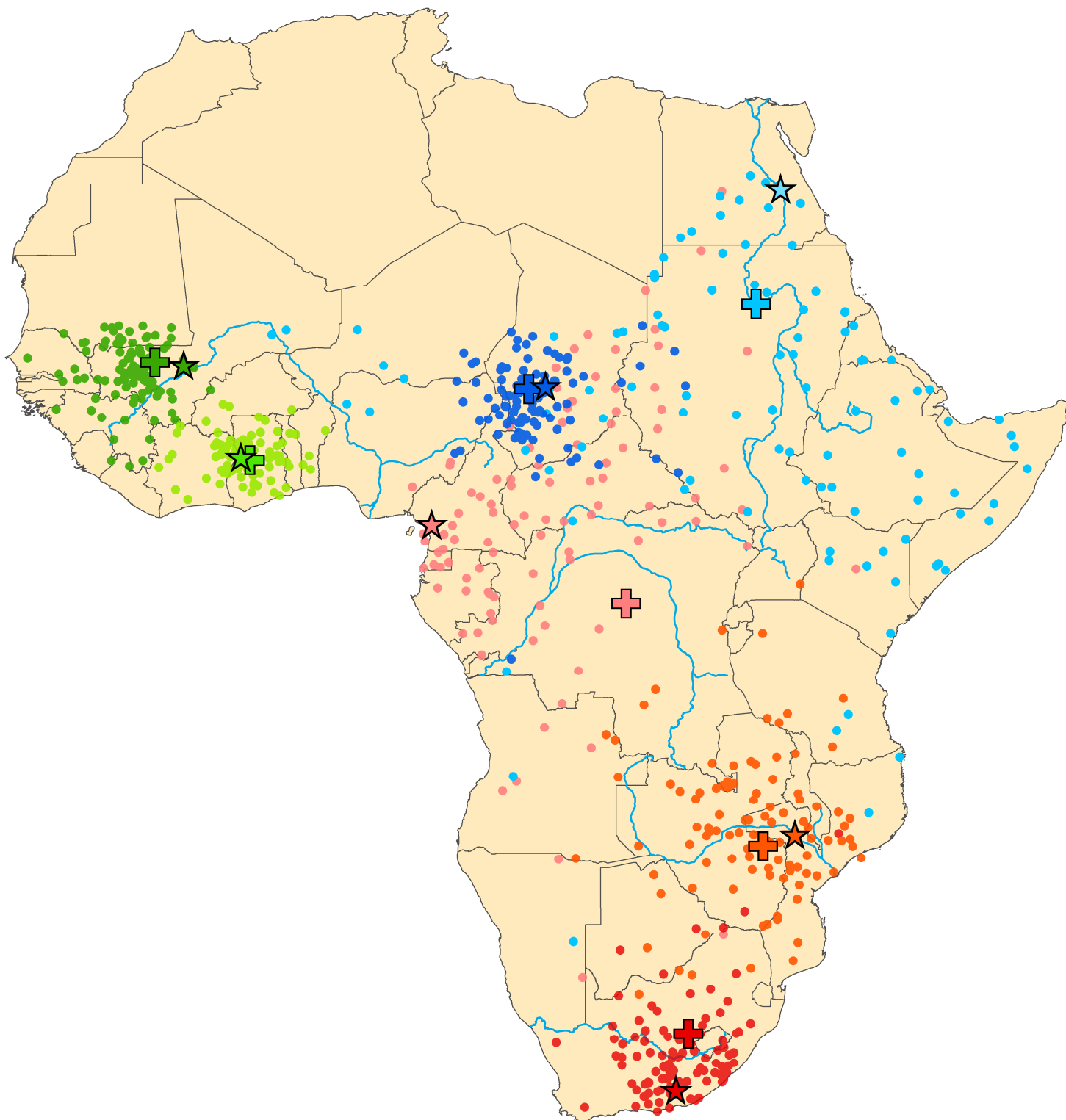

0 750 1,500 3,000 Km

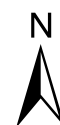

Supplement: Supplemental file 5 Continuous Assignment Method (CAM) results showing select Varanus niloticus reference individuals from each subclade, delineated by differing colors. Stars represent the actual locality and crosses mark the median estimated locality, averaged across 10 runs. The surrounding point [file rsos150619supp5.pdf]

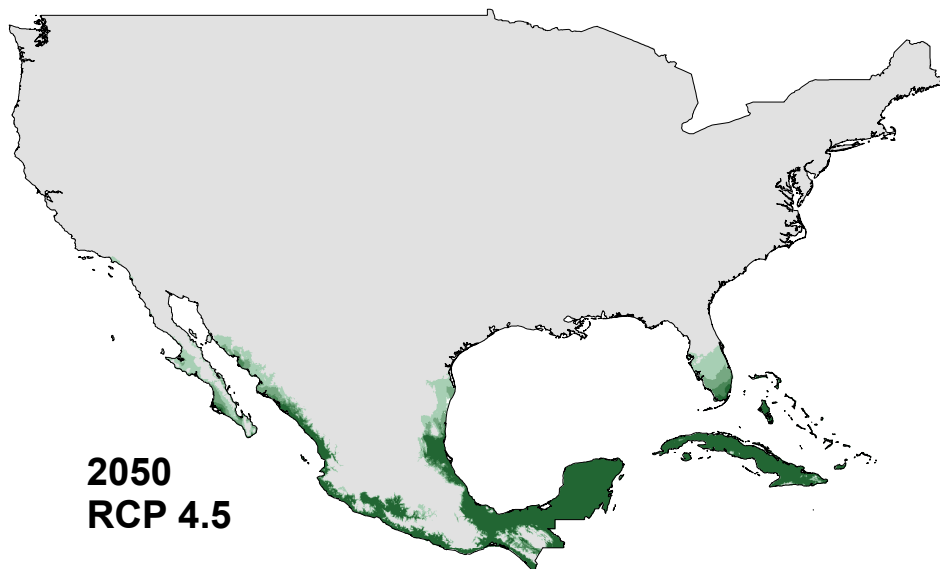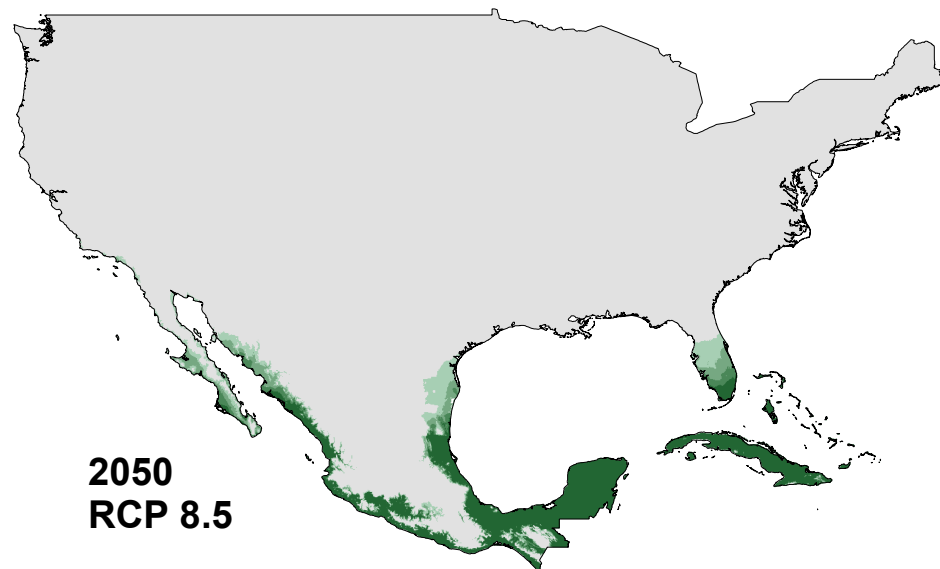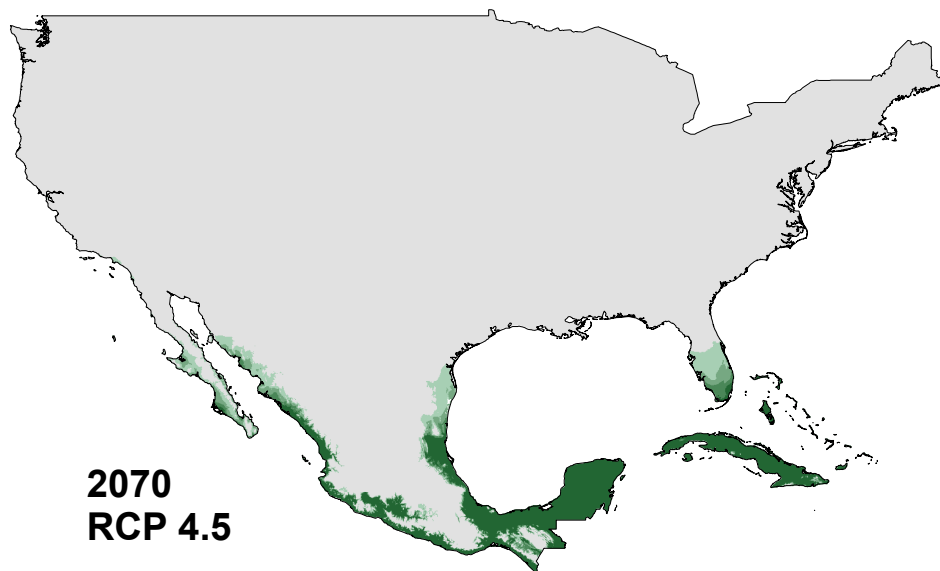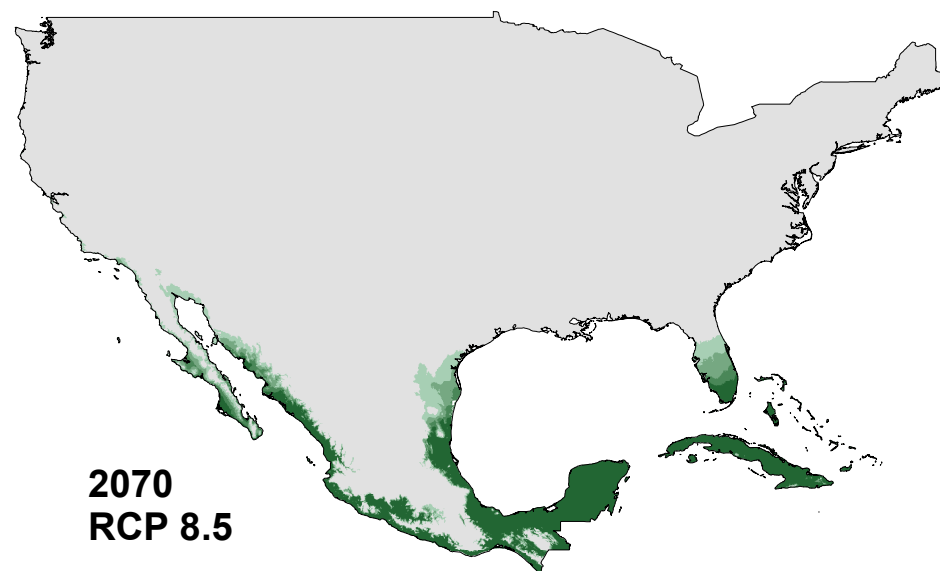

0 500 1,000 2,000 Km

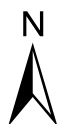

### Suitability

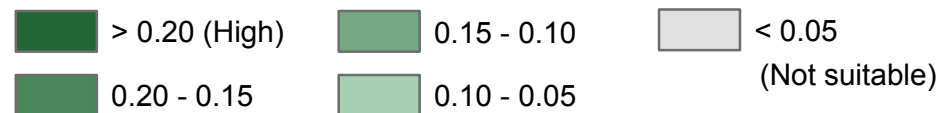

Supplement: Supplemental file 6 Ecological niche model (ENM) of the Varanus niloticus source population showing future climate projections with the National Center for Atmospheric Research's Community Climate System Model (CCSM4) for the years 2050 and 2070. Representative Concentration Pathways (RCPs) of 4.5 ( [file rsos150619supp6.pdf]

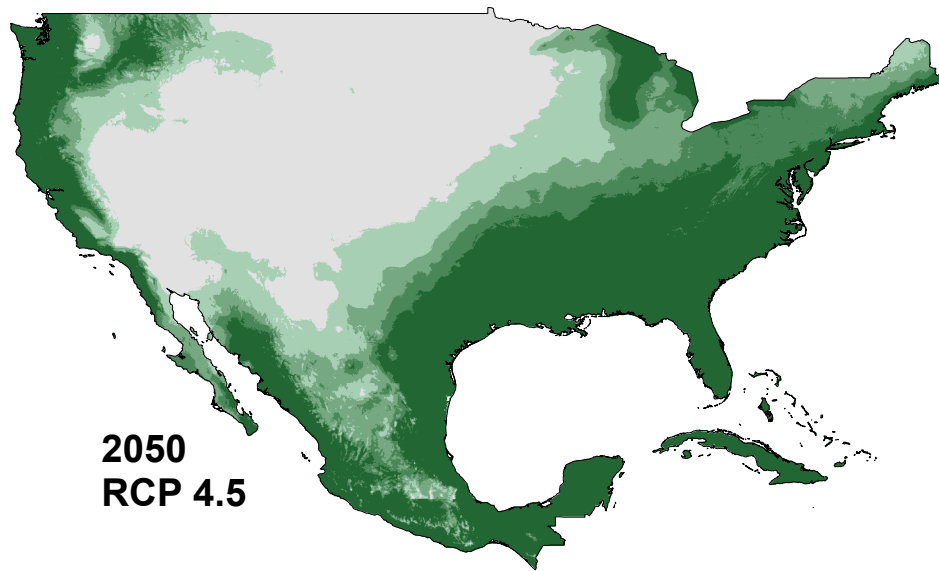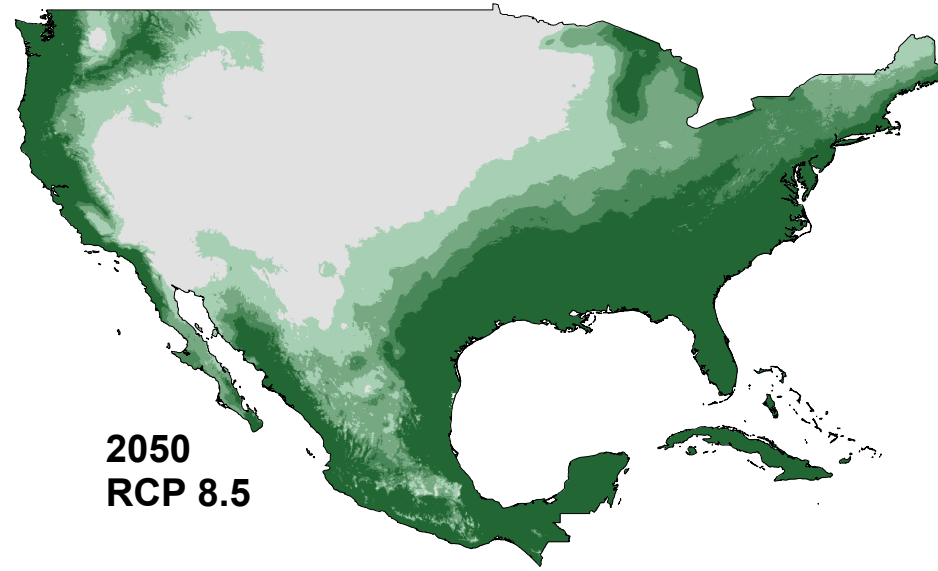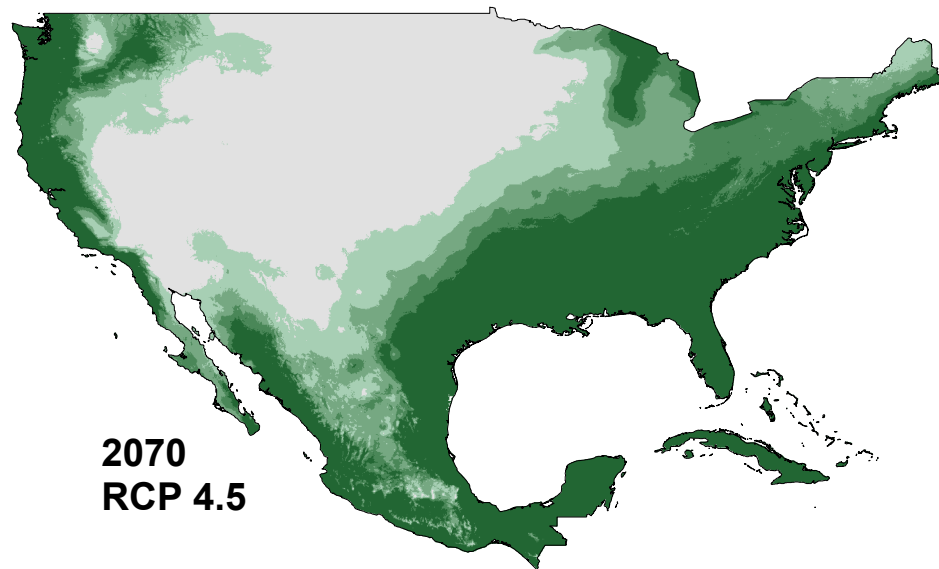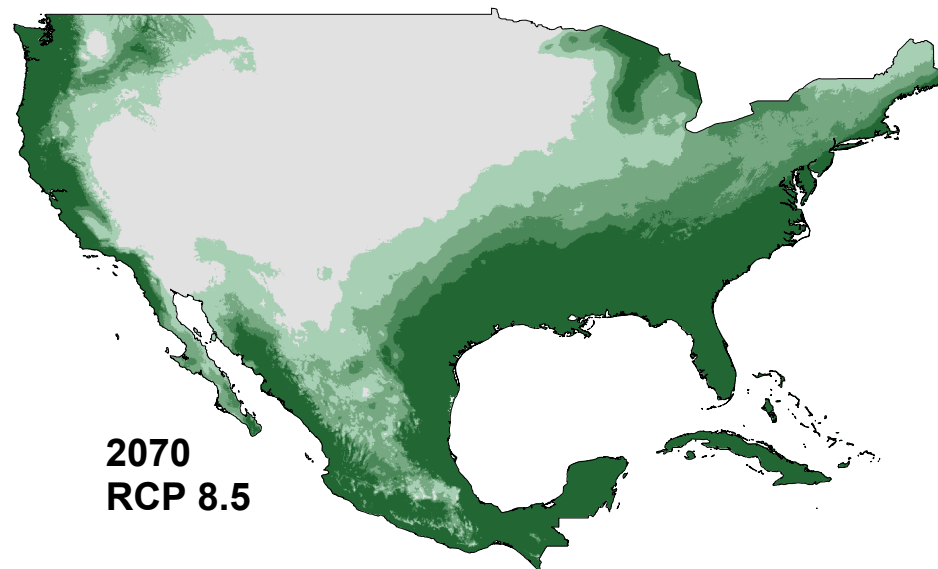

0 500 1,000 2,000 Km

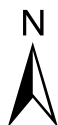

### Suitability

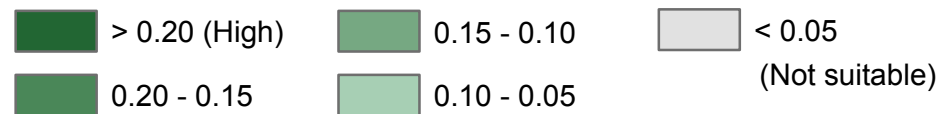

Supplement: Supplemental file 7 Ecological niche model (ENM) of the full Varanus niloticus distribution showing future climate projections with the National Center for Atmospheric Research's Community Climate System Model (CCSM4) for the years 2050 and 2070. Representative Concentration Pathways (RCPs) of 4.5 ( [file rsos150619supp7.pdf]
