# Supplementary material for: Combining genetic and distributional approaches to sourcing introduced species: a case study on the Nile monitor (Varanus niloticus) in Florida
Source: R Soc Open Sci. 2016 Apr 20;3(4):150619. doi: 10.1098/rsos.150619 (PMC4852627; doi:10.1098/rsos.150619)
Supplement: Supplemental Material Legends [file rsos150619supp8.docx]

**Supplemental Material Legends**

**Supplemental file 1** Table of locality and collector information for all *Varanus niloticus* reference individuals and introduced populations

**Supplemental file 2** Spreadsheet of microsatellite genotypes for reference and Florida *Varanus niloticus* individuals

**Supplemental file 3** GPS coordinates in the boundary file used for the continuous assignment method (CAM) in SCAT

**Supplemental file 4** Maximum likelihood tree in Newick format

**Supplemental file 5** Continuous Assignment Method (CAM) results showing select *Varanus niloticus* reference individuals from each subclade, delineated by differing colors. Stars represent the actual locality and crosses mark the median estimated locality, averaged across 10 runs. The surrounding point coordinates show the confidence in the estimated locality, with geographically clustered points indicating high confidence and more diffuse points representing low confidence. These point coordinates were compiled across all runs and 100 were selected based on the highest log likelihood scores

**Supplemental file 6** Ecological niche model (ENM) of the *Varanus niloticus* source population showing future climate projections with the National Center for Atmospheric Research’s Community Climate System Model (CCSM4) for the years 2050 and 2070. Representative Concentration Pathways (RCPs) of 4.5 (low-moderate climate scenario) and 8.5 (most extreme scenario) are shown

**Supplemental file 7** Ecological niche model (ENM) of the full *Varanus niloticus* distribution showing future climate projections with the National Center for Atmospheric Research’s Community Climate System Model (CCSM4) for the years 2050 and 2070. Representative Concentration Pathways (RCPs) of 4.5 (low-moderate climate scenario) and 8.5 (most extreme scenario) are shown
